# Nutritional Status Impairment Due to Neoadjuvant Chemotherapy Predicts Post-Radical Cystectomy Complications

**DOI:** 10.3390/nu13124471

**Published:** 2021-12-14

**Authors:** Sharon Cohen, Jonathan Gal, Yuval Freifeld, Sobhi Khoury, Yoram Dekel, Azik Hofman, Kamil Malshi, Gilad Amiel, Itay Sagi, Ilan Leibovici, Shay Golan, Jack Baniel, Barak Rozenzweig, Zohar Dotan, Miki Haifler

**Affiliations:** 1 Department of Urology, Chaim Sheba Medical Center, Tel Hashomer, Ramat Gan 5266202, Israel; sdfsdf@gmail.com (S.C.); sdrfsdf@gmail.com (B.R.); sdfsdzxc@gmail.com (Z.D.); 2 Sackler Faculty of Medicine, Tel Aviv University, Tel Aviv 6997801, Israel; sdafsdf@gmail.com (J.G.); sZXdfsdf@gmail.com (I.S.); sdZXCfsdf@gmail.com (I.L.); sqdfsdf@gmail.com (S.G.); sdfsrdf@gmail.com (J.B.); 3 Department of Urology, Shamir Medical Center, Tzrifin 7073001, Israel; 4 Department of Urology, Carmel Medical Center, Haifa 3436212, Israel; sacdfsd@gmail.com (Y.F.); sdwfsdf@gmail.com (S.K.); sdfwwsdf@gmail.com (Y.D.); 5 Rappaport Faculty of Medicine, Technion, Israel Institute of Technology, Haifa 3109601, Israel; sdfasdf@gmail.com (A.H.); sdfXsdf@gmail.com (K.M.); sdfqsdf@gmail.com (G.A.); 6 Department of Urology, Rambam Medical Center, Haifa 3109601, Israel; 7 Israel Urologic Oncology Collaboration, Petah-Tikva 4941492, Israel; 8 Department of Urology, Meir Medical Center, Kfar Sab 4428164, Israel; 9 Department of Urology, Rabin Medical Center, Petah-Tikva 4941492, Israel

**Keywords:** radical cystectomy, complication, sarcopenia, nutrition

## Abstract

Background: Radical cystectomy (RC) is the standard treatment for muscle invasive bladder cancer (MIBC). Neoadjuvant chemotherapy (NAC) is associated with improved patient survival. The impact of NAC on nutritional status is understudied, while the association between malnutrition and poor surgical outcomes is well known. This study aims to examine the association between NAC, nutritional status impairment, and post-operative morbidity. Materials and Methods: We included MIBC patients who underwent RC and received NAC from multiple academic centers in Israel. Cross-sectional imaging was used to measure the psoas muscle area and normalized it by height (smooth muscle index, SMI). Pre- and post-NAC SMI difference was calculated (represents nutritional status change). The primary outcomes were post-RC ileus, infection, and a composite outcome of any complication. Logistic regression models were fit to identify independent predictors of the outcomes. Results: Ninety-one patients were included in the study. The median SMI change was −0.71 (−1.58, −0.06) cm^2^/m^2^. SMI decline was significantly higher in patients with post-RC complications (−18 vs. −203, *p* < 0.001). SMI change was an independent predictor of all complications, ileus, infection, and other complications. The accuracy of SMI change for predicting all complications, ileus, infection, and other complications was 0.85, 0.87, 0.75, and 0.86, respectively. Conclusions: NAC-related nutritional deterioration is associated with increased risk of complications after RC. Our results hint towards the need for nutritional intervention during NAC prior to RC.

## 1. Introduction

Bladder cancer is the most lethal neoplasm of the urinary tract with just over 81,000 patients diagnosed every year, 25% of which are muscle invasive (MIBC) at diagnosis [1]. According to the European Association of Urology clinical guidelines, radical cystectomy (RC) with pelvic lymph node dissection is the standard of care for nonmetastatic MIBC. Despite this radical approach, only 50% of the patients survive more than 5 years after diagnosis. To improve these unsatisfactory results, neoadjuvant chemotherapy (NAC) has been used since the 1980s [2] and has proven effective in several randomized controlled trials [3]. The link between chemotherapy and poor nutritional status has been well documented in the scientific literature [4,5,6]. About 40% of all patients receiving anticancer treatment are malnourished [7], and 50–80% of cancer patients are cachectic [8]. Malnutrition is also associated with poor prognosis, decreased response to therapy, prolonged/enhanced treatment morbidity, and decreased quality of life [9]. The impact of platinum-based NAC on nutritional status is understudied, and the results of the existing studies are controversial. In a study on pancreatic cancer patients, retinol-binding protein, pre-albumin, neutrophil-to-lymphocyte ratio, platelet-to-lymphocyte ratio, and prognostic nutrition index were significantly worse in patients receiving NAC compared to the control group [10]. However, a study examining the effect of NAC on nutritional status in patients with locally advanced gastric cancer showed no difference in nutritional biomarkers between the NAC and control groups [11]. Finally, Glaser et al. examined 38 patients with advanced ovarian cancer who received NAC. The mean serum albumin increased significantly after NAC. Furthermore, pre- and post-NAC computerized tomography assessment revealed improvement in body composition measurements [12]. RC is a morbid operation, commonly followed by ileus (2–32%), infectious (5–39%), and urinary diversion-related (22–33%) complications [13]. Furthermore, the association between malnutrition and poor surgical outcomes is well known. Several studies have shown that the presence of malnutrition biomarkers (e.g., low albumin, BMI, weight loss) increases the likelihood of post-RC complications [14,15,16,17]. In addition, severe wasting of skeletal muscle (sarcopenia) is predictive of oncologic and functional outcomes after RC [4,8,18,19]. Sarcopenia is present in 20–70% of cancer patients, depending on the cancer type. The presence of sarcopenia has been linked to increased drug toxicities, high susceptibility to general medical conditions and frailty, all of which may affect post-RC course [8].

To date, no studies are linking NAC-associated nutritional impairment and post-RC morbidity. In a pilot study, our group showed that NAC has a detrimental effect on smooth muscle mass in MIBC patients and the association of this effect and post-RC morbidity [20]. This study aims to examine the association between NAC before RC, poor nutritional status, and post-operative morbidity.

## 2. Materials and Methods

We retrospectively reviewed the records of MIBC patients who underwent RC and received NAC between 2015–2019 in 5 academic medical centers in Israel. Patients who were missing clinical or imaging data were excluded from the study. Institutional review board approval was obtained in each institute. Due to the retrospective nature of the study, a waiver for informed consent was obtained.

### 2.1. Extracted Variables

Clinicopathological variables including age, gender, BMI (in kg/m^2^), Charlson comorbidity index, American Society of Anesthesiologists (ASA) score, smoking history, NAC regimen, pathological tumor, and lymph node stage were extracted from patients’ electronic records.

**Figure 1:**
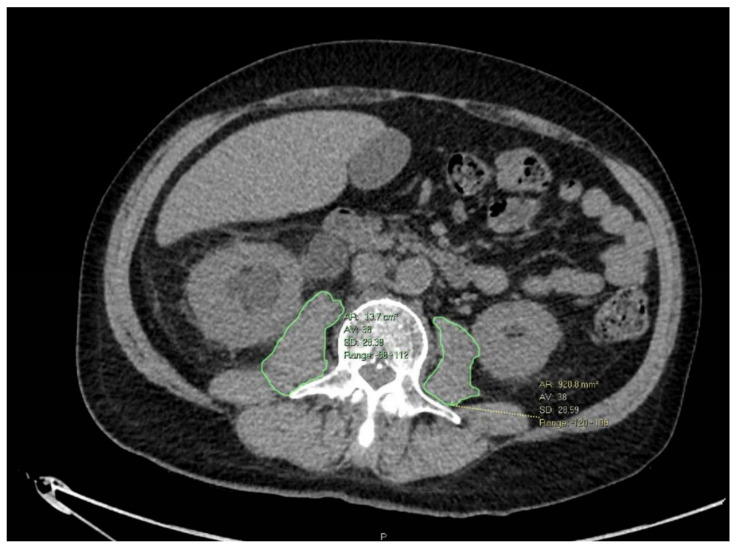
PMA measurements at the L3 vertebrae.

Cross-sectional imaging (computerized tomography (CT) or magnetic resonance imaging (MRI)) was used to measure the psoas muscle area (PMA). Each patient was imaged with one modality throughout his clinical course (either CT or MRI). Pre-NAC imaging was performed within 1 month of starting NAC while post-NAC imaging was performed within 1 month after finishing NAC. Psoas muscle was identified at the level of the third lumbar vertebrae (L3) on which both transverse processes were fully observed. PMA was measured by marking an area of interest around both muscles (Figure 1). Final PMA was calculated as the arithmetic average of both PMAs. Smooth muscle index (SMI) was calculated by normalizing PMA by patient height and reported as cm^2^/m^2^, according to the convention for body composition measurements. Since muscle mass can differ according to gender, age, etc., we examined the SMI difference between pre- and post-NAC (which represented the change in nutritional status) and used it as a predictor variable.

### 2.2. Outcomes

The primary outcomes of interest were post-RC ileus, infection, rehospitalization, other complications and a composite outcome of any complication. We defined complication as any deviation from the normal post-operative course, as decided by the treating physician) within 1 month after RC. Ileus was defined as lack of bowel movement or flatus > 4 days post-operatively. Infection was defined as sepsis (severe inflammatory response syndrome with an established site of infection).

### 2.3. Statistical Analysis

Categorical variables were summarized with frequency counts (percentage) and continuous variables with medians (interquartile range, IQR). Wilcoxon rank-sum and Fisher’s exact tests were used to compare continuous and categorical features across complication groups, respectively. Uni and multivariable logistic regression models were fit to identify independent predictors of the outcomes (using odds ratios (OR) and 95% confidence intervals (95% CIs)). In order to develop the most parsimonious multivariable models all features with a *p*-value < 0.25 on univariable analysis were included in the final multivariable models as previously described [21]. Sample size estimation was based on data from a preliminary study including 40 patients [20]. The effect size in this study was McFadden-R^2^ = 0.29, considered to be medium using Cohen’s (1988) criteria [22]. For the purpose of multiple regression analysis, with a McFadden-R^2^ = 0.2, alpha = 0.05 and power = 0.80, the minimal sample size is approximately *n* = 69 patients [23]. We proposed a sample size of *n* = 90, which will be more than adequate for the main objective of this study. Statistical analyses were performed using the R version 3.6.1: R Foundation for Statistical Computing (Vienna, Austria). All tests are 2 sided, with a *p*-value < 0.05 considered to be statistically significant.

## 3. Results

**Figure 2:**
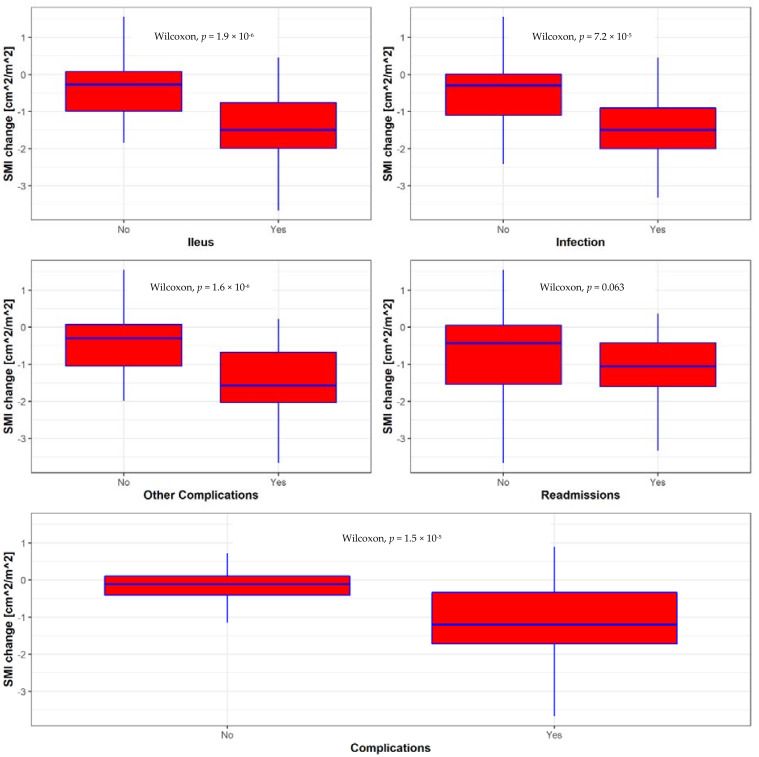
SMI change during NAC across the complication groups. SMI change was significantly lower in patients with complications.

Ninety-one patients were eligible to be included in the study (Table 1). Most patients (73%) were males and had prior smoking history (55%). The median age was 67 (60.74) years. The most common chemotherapy regimen was Gemcitabine–Cisplatin (GC) (54%) followed by Methotrexate–Vinblastine–Adriamycin–Cisplatin (MVAC) (27%). Sixty-three (69%) patients experienced at least one complication after RC, with ileus being the most common (42 (46%) patients). Median BMI was 28 kg/m^2^. Fifty-five (60.4%) and four (4.3%) patients had BMI > 27 and <18, respectively. Sixty-nine (75.8%) patients experienced PMA reduction over the NAC course and the median pre-NAC PMA and PMA changes were 1195 (975, 1420) and −122 (−272, −10) mm^2^, respectively. The median changes in SMI and BMI after NAC were −0.71 (−1.58, −0.06) cm^2^/m^2^ and −1 (−2.7, 1) kg/m^2^, respectively. There was no difference in gender, age, CCI, ASA score, smoking status, and pre-NAC BMI across the complication groups. Patients receiving MVAC had significantly higher complication rates compared with GC (88 vs. 51%, *p* = 0.045). Pre-NAC SMI and SMI decline were significantly higher in patients with post-RC complications (7.23 vs. 6.64 mm^2^, *p* = 0.045 and −1.2 vs. −0.11, *p* < 0.001, respectively). There was no difference in BMI change across these groups (−0.9 vs. −1, *p* = 0.9). Similar results were obtained for patients with and without ileus (Table 2). Both NAC regimen and pre-NAC SMI were similar among patients with and without infection, while the SMI change was significantly higher in patients experiencing an infection (−1.5 vs. −0.3, *p* < 0.001). Finally, pre-NAC SMI and SMI changes were significantly different between patients with and without other complications (6.8 vs. 7.7, *p* = 0.037 and −1.57 vs. −0.3, *p* < 0.001, respectively, Table 3). None of these parameters were significantly different across readmission groups. There was no difference in SMI or BMI change between patients with or without readmission (−0.42 vs. −1.06 (*p* > 0.9) and −1 vs. −1 (*p* > 0.9), respectively, sup Table 1). SMI change across the different complication groups is depicted in Figure 2.

**Figure 3:**
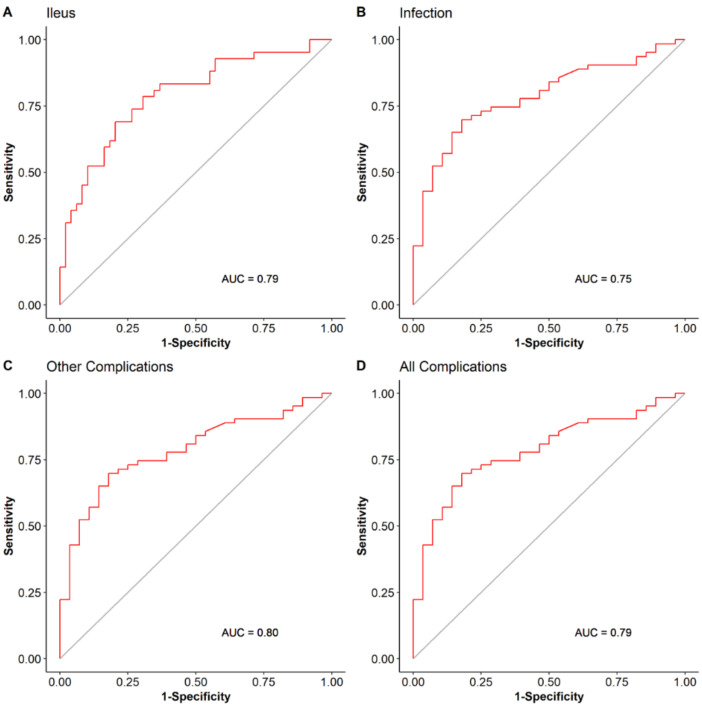
SMI change accuracy in predicting complications in univariable models.

**Figure 4:**
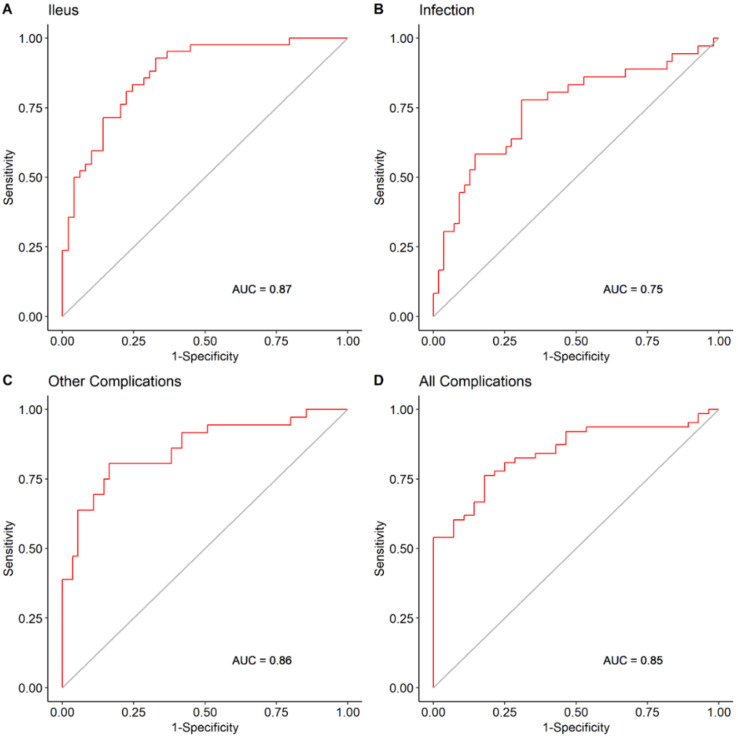
SMI change based multivariable models’ accuracy in predicting complications.

On univariable analysis, SMI change was an independent predictor of all complications, ileus, infection, and other complications (Table 4). This predictive ability was maintained in the multivariable analysis as well. All other clinical variables included in the multivariable model were not independently associated with the examined outcomes (Table 5). The ROC curve AUC of SMI change for predicting all complications was 0.79 with the univariable model (Figure 3), however, it increased to 0.87 with the multivariable model (Figure 4). A similar increase in predictive accuracy with the multivariable models was observed for ileus, infection, and other complications. SMI change and all other clinical variables did not predict readmissions (OR 0.7 (0.45, 1.07), *p* = 0.5, Supplementary Material). BMI change was not an independent predictor of any of the univariable study outcomes and therefore was not entered into a multivariable model (Table 3).

## 4. Discussion

RC is a morbid procedure with a 28–64% complication rate, 27% readmission rate, and 3–7% mortality rate in the first 90 days post-surgery [16]. The association between nutritional status and post-RC complications is well known. Johnson and colleagues used National Surgical Quality Improvement Program (NSQIP) data to retrospectively assess the association of nutritional markers and post-RC complications. Albumin dichotomized to </>3.5 and as a continuous variable were independent predictors of post-RC complications (OR 1.79 (1.06, 3.03) and 1.20 (1.02, 1.40), respectively) [16]. Arora et al. retrospectively examined more than 2000 RC cases from the NSQIP database and demonstrated a significant association between hypoalbuminemia and post-RC complications and mortality [24]. Garg et al. examined 1097 RC cases and demonstrated a 39 and 67% decrease in complication and mortality rates for each increase of one unit of albumin, respectively [25]. Finally, Allaire et al. performed a prospective evaluation of nutritional factors’ effect on post-RC complications. The authors identified Albumin < 3.5 as a predictor of 30 days post-RC high-grade complications (RR 5.52 (1.36–22.35)). In addition, high BMI (>27) was an independent predictor of post-RC low-grade complications (RR 1.33 (1.00–1.77)). In Johnson et al.’s study, BMI and weight loss were not identified as complication predictors (OR 1.01 (0.98, 1.04) and 1.05 (0.44, 2.52), respectively) [16]. Gregg et al. examined the effect of different nutritional parameters on mortality in 538 RC patients. The only significant factor on the multivariable model was albumin while omitting BMI from the model did not significantly change the hazard ratios. The authors concluded that albumin is a sufficient parameter for nutritional risk stratification pre-RC [26]. Finally, sarcopenia was also examined as a nutritional marker for mortality and complications after RC. Psutka et al. used specialized software and a standard definition of sarcopenia to assess its effect on RC patients. The prevalence of sarcopenia in the study’s cohort was 68%. While BMI was not an independent predictor, sarcopenia was highly associated with mortality (HR 2.2 [1.2–3.6]) [18]. Smith et al., defined sarcopenia as low psoas muscle cross-sectional area and found it was significantly associated with post-RC complications (OR 2.25 [1.11, 4.56] [19]. The above-mentioned studies examined the nutritional effect on post-RC complications. However, they did not account for the effect of NAC on the patient’s nutritional status. While the effect of induction chemotherapy on nutritional status has been investigated, the effect of NAC is still controversial. Naumann et al. demonstrated that neoadjuvant chemoradiation in pancreatic cancer patients caused significant weight loss but did not influence survival [27]. Tashiro et al. randomized pancreatic cancer patients before pancreaticoduodenectomy to NAC and no NAC groups. Several nutritional parameters (e.g., pre-albumin, neutrophil to lymphocyte ratio) were significantly worse in the NAC group compared to the no NAC group. There was no significant increase in the incidence of post-operative complications in the NAC group [10]. To our knowledge, there is no study examining the effect of NAC on nutritional status in MIBC patients and its association with post-RC complications. Sarcopenia is caused by both nutritional deficit and systemic stress and is an ongoing process. We hypothesized that the nutritional state dynamics is more influential on outcomes than a single time point value. Our study clearly demonstrates the detrimental effect of NAC on nutritional status of MIBC patients as measured by sarcopenia. The median change in PMA was −122 (−272, −10) mm^2^, which constitutes more than a 10% decline in muscle mass compared to the pre-NAC period. Even after normalizing for height, SMI demonstrated more than a 10% decline. Furthermore, more than 75% of patients had a SMI decline over the NAC course, highlighting the detrimental nutritional effect of chemotherapy. Interestingly, the pre-NAC SMI of patients with all complications, ileus, and other complications in our study was higher than that of patients without these complications (Table 2 and Table 3). These findings hint that initial nutritional status may not be the significant factor in affecting future complications but rather the nutritional status dynamics is more influential. Our multivariable models support this point as pre-NAC SMI was not an independent predictor of post-RC complications (Table 5). We are the first to demonstrate the association of SMI dynamics with morbidity after RC. We hypothesize that changing the SMI dynamics during NAC may lower the complication rate after RC. This may be achieved by a nutritional intervention during NAC administration. Post-NAC SMI values were similar across different complication groups and did not predict any of the complications in this study. Post-NAC SMI is the equivalent of sarcopenia in other studies and our negative result may be explained by the different methodology of calculating SMI. Several previous studies demonstrated the association of sarcopenia with complications [18,19], but these studies defined sarcopenia as the muscle mass normalized by height before RC at a specific point, thus not differentiating between patients with inherent low muscle mass to those losing it during NAC. We believe that these patient populations are different according to our results. Most of the included patients in our study had high pre-NAC BMI, but it was not associated with post-RC complications. Furthermore, BMI change was not an independent predictor of complications. Our results are similar to Gregg and Johnson’s studies which also did not identify any such association [16,26] and opposite to Allaire’s study that demonstrated the association between high BMI and low-grade complications after RC. These conflicting results underscore the complex nature of BMI as an indicator of nutritional status [15].

Our study has several limitations. First, the retrospective design may cause unknown confounders to influence the results. For example, mental state in cancer patients is known to affect their nutritional status and is associated with complications. However, our study has generated a hypothesis that may, in the near future be examined in a controlled study. Additionally, we did not have access to other nutritional or frailty features such as albumin, walking speed, or hand grip strength. We did, however, examine two performance status indices (CCI, ASA) that were not independently associated with complications in the multivariable models. Furthermore, we accurately measured muscle wasting by cross-sectional imaging, which is acknowledged as an accurate index of frailty [28]. Finally, the cohort size may seem small which may put our results in controversy. However, we performed a rigorous power analysis based on a preliminary study [20] that allows us to draw reasonable conclusions with the included sample size.

## 5. Conclusions

Patients undergoing NAC before RC experience muscle wasting, as evidenced by SMI decline. In turn, this SMI decline accurately predicts complications after RC. Our results raise the hypothesis that nutritional intervention during NAC may mitigate the nutritional detrimental effects and may lower the rates of post-RC complications. Prospective studies examining this hypothesis are warranted.

## Figures and Tables

**Table 1:** Descriptive statistics of study cohort.

Characteristic ^1^	
**Gender** [*n*]	
Female	25 (27%)
Male	66 (73%)
**Age** [year]	67 (60, 74)
**CCI**	6 (4, 9)
**ASA**	2 (1, 3)
**Smoking** [*n*]	50 (55%)
**NAC regimen** [*n*]	
GC	49 (54%)
MVAC/ddMVAC	25 (27%)
Other	17 (19%)
**RC type** [*n*]	
Open	61 (67%)
RARC	30 (33%)
**Complication type** [*n*]	
All Complications	63 (69%)
Ileus	42 (46%)
Readmissions	30 (33%)
Infection	36 (40%)
Other Complications	36 (40%)
**Pre NAC SMI [cm^2^/m^2^]**	6.96 (5.73, 8.33)
**Post-NAC SMI [cm^2^/m^2^]**	6.17 (4.97, 7.40)
**SMI change [cm^2^/m^2^]**	−0.71 (−1.58, −0.06)
**Pre NAC BMI [kg/m^2^]**	28.0 (25.2, 32.0)
**Post-NAC BMI [kg/m^2^]**	26.9 (23.3, 31.4)
**BMI change [kg/m^2^]**	−1.0 (−2.7, 1.0)

^1^*n* (%); Median (IQR).

**Table 2:** Descriptive statistics according to all complications and ileus.

Characteristic	Any Complication		*p*-Value ^2,3^	Ileus		*p*-Value ^2,3^
	No, *n* = 28 ^1^	Yes, *n* = 63 ^1^		No, *n* = 49 ^1^	Yes, *n* = 42 ^1^	
**Gender [*n*]**			0.7			>0.9
Female	9 (32%)	16 (25%)		14 (29%)	11 (26%)	
Male	19 (68%)	47 (75%)		35 (71%)	31 (74%)	
**Age [year]**	68 (61, 77)	67 (60, 74)	0.6	68 (60, 76)	66 (60, 73)	0.5
**CCI**	6 (4, 51)	6 (4, 8)	0.6	5 (4, 43)	6 (4, 8)	>0.9
**ASA**	2 (1, 3)	2 (1, 3)	0.9	2 (1, 3)	2 (1, 2)	**0.058**
**Smoking [*n*]**	19 (68%)	31 (49%)	0.3	30 (61%)	20 (48%)	0.3
**NAC regimen [*n*]**			**0.045**			**0.006**
GC	21 (49%)	28 (51%)		35 (71%)	14 (29%)	
MVAC/ddMVAC	3 (12%)	22 (88%)		8 (32%)	17 (68%)	
Other	4 (23%)	13 (77%)		6 (35%)	11 (65%)	
**RC type [*n*]**			0.4			0.6
Open	16 (57%)	45 (71%)		31 (63%)	30 (71%)	
RARC	12 (43%)	18 (29%)		18 (37%)	12 (29%)	
**Pre NAC SMI [cm^2^/m^2^]**	6.64 (5.59, 7.35)	7.23 (5.95, 8.66)	**0.045**	6.79 (5.61, 7.50)	7.70 (6.46, 8.96)	**0.031**
**Post-NAC SMI [cm^2^/m^2^]**	6.30 (5.53, 7.08)	6.04 (4.91, 7.64)	>0.9	6.14 (5.15, 7.16)	6.38 (4.81, 7.58)	>0.9
**SMI change [cm^2^/m^2^]**	−0.11 (−0.41, 0.1)	−1.2 (−1.72, −0.34)	**<0.001**	−0.27 (−0.99, 0.08)	−1.50 (−2.00, −0.76)	**<0.001**
**Pre NAC BMI [kg/m^2^]**	27.6 (23.9, 30.7)	29.0 (25.4, 32.0)	0.6	27.3 (24.0, 30.7)	29.1 (26.0, 32.9)	0.11
**Post-NAC BMI [kg/m^2^]**	25.4 (22.8, 29.5)	27.1 (23.6, 31.9)	0.4	26.1 (22.9, 29.4)	28.0 (23.9, 32.4)	0.12
**BMI change [kg/m^2^]**	−0.9 (−3.1, 0.7)	−1.0 (−2.5, 1.2)	0.9	−0.8 (−2.3, 1.1)	−1.5 (−3.0, 0.7)	0.6

^1^ Median (IQR); *n* (%), ^2^ Wilcoxon rank sum exact test; Fisher’s exact test; Wilcoxon rank sum test; ^3^ false discovery rate correction for multiple testing.

**Table 3:** Descriptive statistics according to infection and other complications.

Characteristic	Infection		*p*-Value ^2,3^	Other Complications		*p*-Value ^2,3^
	No, *n* = 55 ^1^	Yes, *n* = 36 ^1^		No, *n* = 55 ^1^	Yes, *n* = 36 ^1^	
**Gender [*n*]**			0.9			0.2
Female	16 (29%)	9 (25%)		19 (35%)	6 (17%)	
Male	39 (71%)	27 (75%)		36 (65%)	30 (83%)	
**Age [year]**	68 (60, 74)	66 (60, 73)	0.9	68 (60, 76)	66 (60, 72)	0.4
**CCI**	6 (4, 26)	6 (4, 8)	0.9	6 (4, 46)	6 (4, 6)	0.2
**ASA**	2 (1, 3)	2 (1, 3)	0.8	2 (1, 3)	2 (1, 3)	0.7
**Smoking [*n*]**	36 (65%)	14 (39%)	**0.086**	38 (69%)	12 (33%)	**0.006**
**NAC regimen [*n*]**			0.8			0.4
GC	32 (65%)	17 (35%)		32 (65%)	17 (35%)	
MVAC/ddMVAC	15 (60%)	10 (40%)		12 (48%)	13 (52%)	
Other	8 (47%)	9 (53%)		11 (65%)	6 (35%)	
**RC type [*n*]**			0.8			**0.011**
Open	35 (64%)	26 (72%)		30 (55%)	31 (86%)	
RARC	20 (36%)	10 (28%)		25 (45%)	5 (14%)	
**Pre NAC SMI [cm^2^/m^2^]**	6.90 (5.58, 8.31)	7.00 (6.37, 8.45)	0.6	6.80 (5.30, 7.66)	7.70 (6.60, 8.69)	**0.037**
**Post-NAC SMI [cm^2^/m^2^]**	6.33 (5.15, 7.28)	5.59 (4.85, 7.51)	0.8	6.14 (4.68, 7.28)	6.38 (5.04, 7.51)	0.8
**SMI change [cm^2^/m^2^]**	−0.30 (−1.10, 0.01)	−1.50 (−2.00, −0.91)	**<0.001**	−0.30 (−1.05, 0.07)	−1.58 (−2.03, −0.68)	**<0.001**
**Pre NAC BMI [kg/m^2^]**	28.8 (25.0, 32.0)	27.9 (25.3, 32.0)	0.9	28.8 (24.6, 32.0)	27.9 (25.9, 30.6)	0.8
**Post-NAC BMI [kg/m^2^]**	27.0 (23.2, 31.2)	26.5 (23.3, 31.4)	>0.9	26.8 (23.0, 31.4)	27.1 (23.9, 31.0)	0.7
**BMI change [kg/m^2^]**	−0.9 (−3.2, 1.2)	−1.7 (−2.3, 0.7)	0.9	−1.0 (−2.9, 0.8)	−1.0 (−2.2, 1.2)	0.8

^1^ Median (IQR); *n* (%); ^2^ Wilcoxon rank sum exact test; Fisher’s exact test; Wilcoxon rank sum test; ^3^ false discovery rate correction for multiple testing.

**Table 4:** Univariable analysis of different complications.

Characteristic	All Complications			Ileus			Infection			Other Complications		
	OR ^1^	95% CI ^1^	*p*-Value	OR ^1^	95% CI ^1^	*p*-Value	OR ^1^	95% CI ^1^	*p*-Value	OR ^1^	95% CI ^1^	*p*-Value
**Gender**			0.5			0.8			0.7			0.056
Female	—	—		—	—		—	—		—	—	
Male	1.39	0.51, 3.66		1.13	0.45, 2.89		1.23	0.48, 3.29		2.64	0.98, 8.01	
**CCI**	0.99	0.98, 1.00	0.2	1	0.99, 1.01	0.6	1	0.99, 1.01	>0.9	0.99	0.97, 1.00	0.076
**ASA**	0.93	0.56, 1.52	0.8	0.58	0.35, 0.93	**0.022**	0.88	0.55, 1.41	0.6	0.88	0.55, 1.41	0.6
**Smoking**	0.46	0.17, 1.14	0.1	0.58	0.25, 1.32	0.2	0.34	0.14, 0.79	**0.012**	0.22	0.09, 0.54	**<0.001**
**NAC Regimen**			**0.014**			**0.001**			0.4			0.3
GC	—	—		—	—		—	—		—	—	
MVAC/	5.5	1.63, 25.4		5.31	1.93, 15.8		1.25	0.46, 3.39		2.04	0.77, 5.52	
ddMVAC												
other	2.44	0.74, 9.63		4.58	1.46, 15.7		2.12	0.69, 6.64		1.03	0.31, 3.21	
**RC type**			0.2			0.4			0.4			**0.001**
Open	—	—		—	—		—	—		—	—	
RARC	0.53	0.21, 1.36		0.69	0.28, 1.66		0.67	0.26, 1.65		0.19	0.06, 0.54	
**Pre NAC SMI**	1.35	1.05, 1.76	**0.045**	1.39	1.11, 1.80	**0.014**	1.17	0.94, 1.47	0.6	1.37	1.09, 1.78	**0.016**
**Pre NAC BMI**	1.04	0.95, 1.15	0.5	1.11	1.01, 1.22	**0.056**	0.99	0.90, 1.09	>0.9	0.99	0.91, 1.09	>0.9
**SMI change**	0.26	0.12, 0.49	**<0.001**	0.28	0.15, 0.48	**<0.001**	0.37	0.21, 0.61	**<0.001**	0.29	0.15, 0.49	**<0.001**
**BMI change**	1.01	0.90, 1.14	0.8	0.97	0.86, 1.08	0.7	1	0.89, 1.12	>0.9	1.02	0.92, 1.15	0.7

^1^ OR = odds ratio; CI = confidence interval; false discovery rate correction for multiple testing.

**Table 5:** Multivariable analysis of different complications.

Characteristic	All Complications			Ileus				Infection		Other Complications		
	OR ^1^	95% CI ^1^	*p*-Value	OR ^1^	95% CI ^1^	*p*-Value	OR ^1^	95% CI ^1^	*p*-Value	OR ^1^	95% CI ^1^	*p*-Value
**Gender**												0.7
Female										—	—	
Male										1.31	0.31, 5.78	
**CCI**	0.98	0.96, 1.00	0.3							0.99	0.97, 1.02	0.4
**ASA**				0.69	0.34, 1.35	0.4						
**Smoking**	0.74	0.20, 2.63	0.8	1.01	0.24, 4.41	>0.9	0.72	0.26, 2.10	0.9	0.60	0.16, 2.24	0.7
**NAC regimen**			0.8			0.2						0.4
GC	—	—		—	—					—	—	
MVAC/ddMVAC	2.64	0.52, 15.9		2.97	0.65, 14.3					0.54	0.11, 2.41	
other	1.63	0.31, 10.0		5.51	0.94, 38.6					0.14	0.02, 0.98	
**RC type**			0.8									0.3
Open	—	—								—	—	
RARC	1.38	0.27, 7.64								0.28	0.03, 1.11	
**Pre NAC SMI**	0.99	0.67, 1.51	>0.9	1.13	0.81, 1.59	0.6	1.00	1.00, 1.00	0.8	0.88	0.58, 1.34	0.7
**SMI change**	0.31	0.12, 0.72	**0.039**	0.34	0.13, 0.79	**0.045**	0.4	0.2, 0.71	**0.004**	0.2	0.07, 0.49	**0.001**
**Pre NAC BMI**				1.18	0.99, 1.38	**0.051**						
**BMI change**		//		0.95	0.80, 1.13	**0.9**						

^1^ OR = odds ratio; CI = confidence interval; false discovery rate correction for multiple testing.

## Data Availability

The data presented in this study are available on request from the corresponding author. The data are not publicly available due to IRB restrictions.

## Supplementary Materials

The following are available online at https://www.mdpi.com/article/10.3390/nu13124471/s1, Table S1: Descriptive statistics according to readmissions.
