# Supplementary material for: Nutritional Status Impairment Due to Neoadjuvant Chemotherapy Predicts Post-Radical Cystectomy Complications
Source: Nutrients. 2021 Dec 14;13(12):4471. doi: 10.3390/nu13124471 (PMC8708207; doi:10.3390/nu13124471)
Supplement: Supplementary file 1 [file nutrients-13-04471-s001.zip › nutrients-1480534-supplementary.pdf]

	Readmissions		p-value[2, 3]
	No, N = 55[1]	Yes, N = 36[1]	
**Gender [n]**			>0.9
Female	17 (28%)	8 (27%)	
Male	44 (72%)	22 (73%)	
**Age [y]**	68 (62, 75)	65 (59, 70)	0.3
**CCI**	6 (4, 46)	6 (4, 6)	0.4
**ASA**	2 (1, 3)	2 (1, 3)	0.7
**Smoking [n]**	36 (59%)	14 (47%)	0.6
**NAC regimen [n]**			0.8
GC	35 (57%)	14 (47%)	
MVAC/ddMVAC	15 (25%)	10 (33%)	
Other	11 (18%)	6 (20%)	
**RC type [n]**			0.6
Open	38 (62%)	23 (77%)	
RARC	23 (38%)	7 (23%)	
**Pre NAC SMI [cm2/m2]**	6.86 (5.61, 8.27)	7.00 (6.04, 8.40)	0.6
**Post NAC SMI [cm2/m2]**	6.28 (4.78, 7.34)	6.03 (5.07, 7.51)	>0.9
**SMI change [cm2/m2]**	-0.43 (-1.54, 0.05)	-1.06 (-1.60, -0.43)	0.3
**Pre NAC BMI [kg/m^2]**	27.7 (25.0, 30.7)	29.4 (25.6, 32.3)	0.4
**Post NAC BMI [kg/m^2]**	26.9 (23.2, 29.8)	27.1 (24.2, 32.4)	0.6
**BMI change [kg/m^2]**	-1.0 (-2.6, 0.8)	-1.0 (-2.6, 1.1)	>0.9

[1]Median (IQR); n (%), [2]Wilcoxon rank sum exact test; Fisher's exact test; Wilcoxon rank sum test, [3]False discovery rate correction for multiple testing.

Supplementary Table S1: Descriptive statistics according to readmissions.
